# A Novel Five-Gene Signature Related to Clinical Outcome and Immune Microenvironment in Breast Cancer

**DOI:** 10.3389/fgene.2022.912125

**Published:** 2022-05-13

**Authors:** Yi Yang, Hong-Li Liu, Yi-Jing Liu

**Affiliations:** Chongqing Key Laboratory of Translational, Research for Cancer Metastasis and Individualized Treatment, Chongqing University Cancer Hospital, Chongqing, China

**Keywords:** breast cancer, immune cell infiltration, biomarker, signature, Kif4A

## Abstract

Breast cancer (BC) is the most frequent cancer in women and the main cause of cancer-related deaths in the globe, according to the World Health Organization. The need for biomarkers that can help predict survival or guide treatment decisions in BC patients is critical in order to provide each patient with an individualized treatment plan due to the wide range of prognoses and therapeutic responses. A reliable prognostic model is essential for determining the best course of treatment for patients. Patients’ clinical and pathological data, as well as their mRNA expression levels at level 3, were gleaned from the TCGA databases. Differentially expressed genes (DEGs) between BC and non-tumor specimens were identified. Tumor immunity analyses have been utilized in order to decipher molecular pathways and their relationship to the immune system. The expressions of KIF4A in BC cells were determined by RT-PCR. To evaluate the involvement of KIF4A in BC cell proliferation, CCK-8 tests were used. In this study, utilizing FC > 4 and *p* < 0.05, we identified 140 upregulated genes and 513 down-regulated genes. A five-gene signature comprising SFRP1, SAA1, RBP4, KIF4A and COL11A1 was developed for the prediction of overall survivals of BC. Overall survival was distinctly worse for patients in the high-risk group than those in the low-risk group. Cancerous and aggressiveness-related pathways and decreased B cell, T cell CD4^+^, T cell CD8^+^, Neutrophil and Myeloid dendritic cells levels were seen in the high-risk group. In addition, we found that KIF4A was highly expressed in BC and its silence resulted in the suppression of the proliferation of BC cells. Taken together, as a possible prognostic factor for BC, the five-gene profile created and verified in this investigation could guide the immunotherapy selection.

## Introduction

Breast cancer (BC) remains to be the most common cancer and the most frequent cause of cancer death in females worldwide (Buja et al., 2020; Hanker et al., 2020). Progress in pathological characterisation and molecular processes research has made it possible to better diagnose and treat BC (Yin et al., 2020). However, morbidity and fatality rates for BC patients have risen by over 20 and 14% since 2008 (Tay and Tan, 2021). Until further notice, the most effective method of preventing and controlling local recurrence of BC is adjuvant chemotherapy and radiotherapy followed by surgery (Eini et al., 2021; Ye et al., 2021). The majority of breast cancer tumors were discovered clinically at an advanced stage despite the fact that considerable efforts were made to improve the detection and treatment, and the disease Karyotypic studies further show that BC gets increasingly aggressive by accumulating genetic alterations in a stepwise manner (Garcia-Martinez et al., 2021; Sivaganesh et al., 2021). Increasing attention has been paid to individualized and accurate therapeutic strategies in the field of clinical treatment. Thus, finding new biomarkers and targets for prognostication is therefore seen as a useful strategy for achieving this objective.

BC is not a single disease, but rather a collection of disorders with a wide range of clinical characteristics, treatment responses, and outcomes, even among individuals who are in the same stage of the disease (De Cicco et al., 2019). Recent advances in “omics” technology have revealed new details about the molecular complexity of BC, inspiring scientists to look for new ways to better identify patients at risk for the disease (Garrido-Castro et al., 2019; Tagliafico et al., 2020). Multigene signatures may be more accurate than conventional risk classification methods in BC, according to a number of studies (Sporikova et al., 2018; Li et al., 2021). For instance, Five-gene prognostic model (KRT6A, E2F7, DCBLD2, ASPM and ADM) derived from the TCGA PAAD dataset and shown to be accurate in predicting overall survival. (Liu et al., 2021). Zhang et al. discovered a novel autophagy-related long noncoding RNA signature in BC patients that may bring new insights into predicting the prognosis of patients with BC (Wu et al., 2021). According to a recent study, a new prognostic model connected with nine ferroptosis-related genes was developed, and the model’s good prediction capacity was confirmed by three databases: ICGC, GEO, TCGA datasets (Liang et al., 2020). Prognostic gene signatures based on Chip sequencing (GEO and TCGA, for example) might uncover more survival-associated genes, which in combination with clinical and pathological factors may be a strong tool for the prediction of the outcomes of BC and tailored treatments (Zhang et al., 2018; Gao et al., 2019; Xu et al., 2020).

In the present study, we identified a novel five-gene signature for patients with BC. Our findings might provide an effective prognostic predictor and a new view for individual treatments of BC patients.

## Materials and Methods

### Patient Data Sets

The TCGA (https://cancergenome.nih.gov/) was used to acquire clinical and pathological data from BC patients. The edgeR software was used to normalize gene expression. In this study, a total of 1097 TCGA female BC patients with mRNA expression profiles were used. BC samples with survival information were included in this study. We employed the negative binomial distribution approach to discover differently expressed genes (DGEs) between BC specimens and non-tumor tissues. The Limma package was applied to perform the analysis (Ritchie et al., 2015). A generalized linear model for each gene is fitted using the Limma package’s negative binomial distribution, and empirical Bayes shrinkage is used to estimate dispersion and fold-change. There were no genes with an average count value of less than 1 that could be included in the raw data set. When |log2 fold change (FC)| >4 and a false discovery rate (FDR) < 0.05 were taken into consideration, we employed Limma program to identify the differentially expressed DGEs. In addition, BC gene expression profiles (GSE7904) were downloaded from the Gene Expression Omnibus (GEO) database (https://www.ncbi.nlm.nih.gov/geo/). GSE7904 dataset included 19 non-tumor specimens and 43 BC specimens.

### GO and KEGG Pathway Analysis

When performing a GO analysis, genes are broken down into their molecular functions, biological processes, and cellular components, all of which are addressed in separate sections of the report. KEGG is a method for analyzing data to determine which biological pathways a set of genes is particularly prominent in. “clusterProfiler” R package was used to perform GO and KEGG pathway analysis based on DEGs between BC specimens and non-tumor specimens (Yu et al., 2012).

### Survival Analysis

The TCGA database has clinical data and related information downloaded, and we now need to gather data on over-survival (OS), eliminating entries for instances for which there are no data. The remaining case data was used for further survival analysis. Our survival experiments focused on the top 20 genes that differed between BC specimens and non-tumor specimens. Assays of survival curves were done by the use of the Kaplan-Meier methods.

### Verification of Genes in GEPIA Database

An online database that utilizes data from the UCSC Xena program is called the Gene Expression Profiling Interactive Analysis (GEPIA, http://gepia.cancer.pku.cn/). It is possible to use the database to look for changes in gene expression between various malignancies and healthy tissues, as well as the overall survival rate, by using the expression analysis and custom data analysis methods. Cancer and healthy tissues can be compared using the GEPIA database to examine expression differences. We used the GEPIA database to confirm the hub gene’s mRNA expression level.

### Construction and Validation of a Prognostic Gene Signature

LASSO penalized Cox regression was used to build a prognostic model following the collection of survival-related DGEs to avoid overfitting (McEligot et al., 2020). Centralization and normalization (using R’s “scale” function) of the TCGA expression data resulted in the risk score being tallied, and the risk score formula was as follows: Risk Score = ∑^7^_i_Xi×Yi (X: coefficients, Y: gene expression level). By comparing the median OS time across low- and high-risk BC subgroups, all patients were classified as either low- or high-risk. The “survival”, “survminer” and “timeROC” R packages were employed.

### Difference of Tumor-Infiltrating Immune Cells in BC

In order to examine the connections between risk score and the infiltration levels of six immune cells (including dendritic cells, macrophases, neurphils, CD8 + T cells, CD4 + T cells and B cells, the public database Tumor Immune Estimation Resource (TIMER) was applied.

### Analyses and Visualization of Somatic Mutations

The Maftools R/Bioconductor software was used to retrieve the mutational data from the MAF file. Following that, the MAF file summary was shown using the plotmafSummary function to show the number of variation types and classifications for each variant. Using the oncoplot tool, the top 10 mutant genes and POLE were plotted using the OncoPlot program. In order to plot POLE’s lollipopPlot, the lollipopPlot function was used.

### Cell Lines and RNA Interference

The American Type Culture Collection provided the human BC cell lines MCF-7, SKBR, BT-20, ZR-75-1, MDAMB-231, and an immortalized breast epithelial cell line MCF-10A. In DMEM, ZR-75-1 and BT20 cells were cultivated at 37°C in a humidified atmosphere of 5% CO_2_, whereas MCF7, MDA-MB-231, and SKBR3 were cultured in RPMI-1640 with 10% FBS, 100 U/mL penicillin, and 100 mg/ml streptomycin.

Sigma-Aldrich provided KIF4A small interfering RNA (si-KIF4A) and a negative control siRNA. As instructed by the manufacturer, cells were transfected using Lipofectamine 2000 (Invitrogen).

### Real-Time Quantitative PCR

Trizol was used to lyse the cells, and chloroform and isopropanol were used to extract the RNA. After determining the RNA concentration, the cDNA (complimentary deoxyribonucleic acid) was reverse-transcribed. ABI 7500 instruments are used for real-time quantitative PCR. GAPDH was measured as an internal control and the 2^−ΔΔCT^ method was employed to determine the relative expression of KIF4A. The primers used were as follows: KIF4A forward, 5′-GAG​CTA​TTT​GCC​GAC​AAG​GC-3′; KIF4A reverse, 5′-GGA​GTT​TGC​AAG​ACC​CAT​GC-3′; GAPDH forward, 5′-AGT​TGC​GTT​ACA​CCC​TTT​CTT​G-3′; GAPDH reverse, 5′-TCA​CCT​TCA​CCG​TTC​CAG​TTT-3′.

### Cell Growth Assay

For the cell growth experiments, 4  ×  10^3^ cells per well were seeded into 96-well plates, with three wells used for each tested group. Cell numbers were evaluated over 5 days using a cell counting kit-8 (CCK-8) (SAB, Laifu Technology, Nanjing, China). A 10 μL volume of CCK-8 reagent was applied to each well, and the plate was incubated at 37°C for 2 h. Subsequently, in each well, using a spectrophotometer, we measured the absorbance at 450 nm for each sample.

### Statistical Analysis

All experiments were performed in triplicate. Statistical analyses were performed using R software v3.5.0, SPSS (R Core Team, Massachusetts, USA) or GraphPad Prism software (GraphPad Software, San Diego, CA, USA). Student’s t-test and one-way ANOVA were respectively employed to evaluate two or multiple groups, for statistical significance. The Kaplan-Meier methods were applied to create the survival curves. *p* < 0.05 was considered statistically significant.

## Results

### Identification of the DGEs Between BC Specimens and Non-Tumor Specimens

**FIGURE 1:**
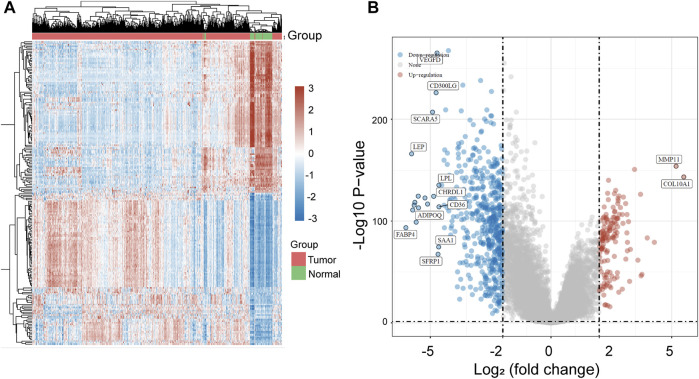
The identification of DGEs in BC based on TCGA datasets. **(A)** Heat map of all DGEs between BC specimens and normal breast specimens. **(B)** Volcanic map of DGEs based on the standard of FC > 4.

To screen possible regulators in BC, we analyzed TCGA datasets using Limma package, and identified many DGEs between BC specimens and normal breast specimens, which were shown in Heat map (Figure 1A). Then, we screened 140 upregulated genes and 513 down-regulated genes using FC > 4 and *p* < 0.05, which were shown in Volcanic map (Figure 1B).

### GO and KEGG Enrichment Analysis

**FIGURE 2:**
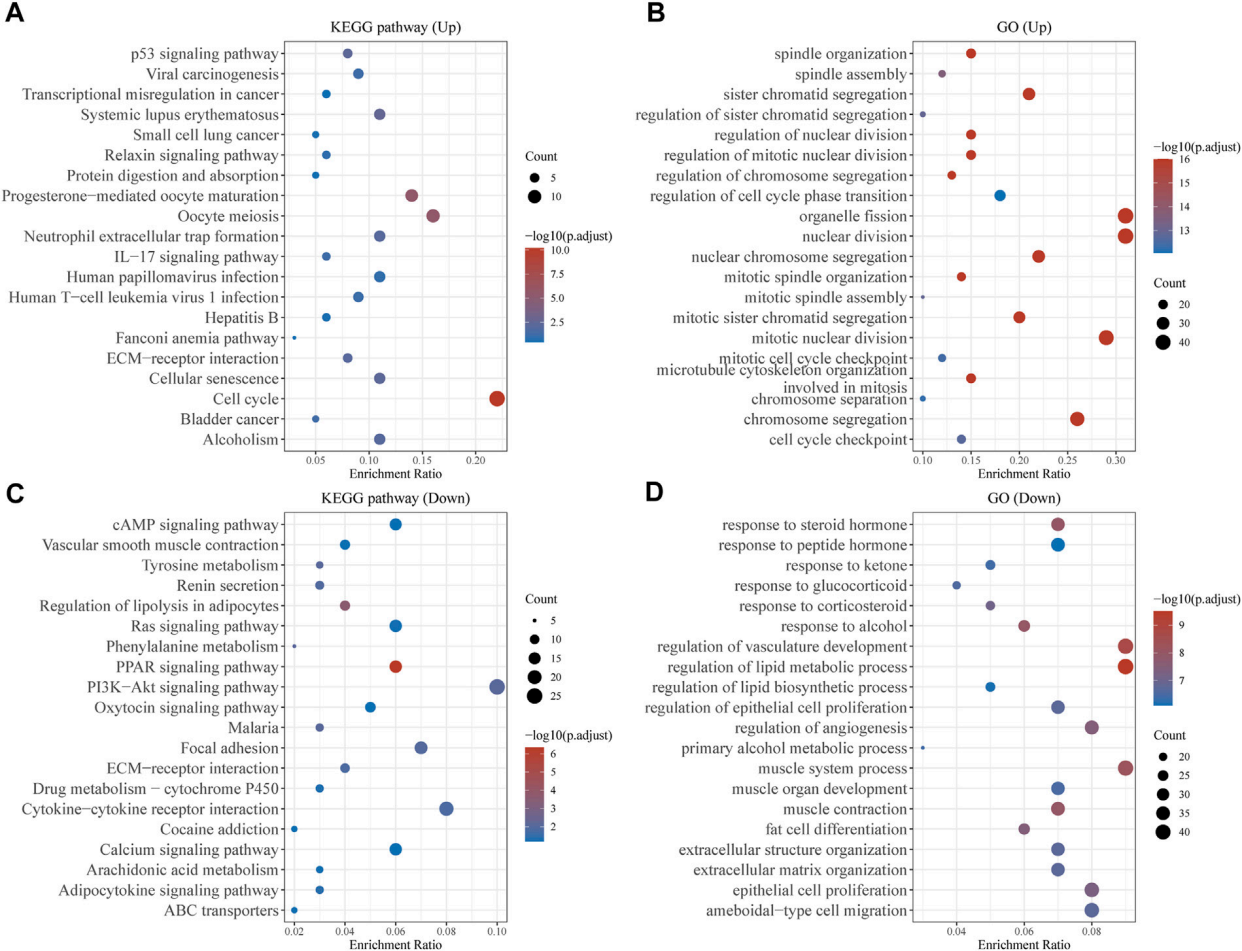
Function Enrichment Analysis of DEGs. **(A,B)** KEGG and G Analysis of 140 upregulated genes in BC. **(C,D)** KEGG and G Analysis of 513 down-regulated genes in BC.

ClusterProfiler was used to undertake enrichment analysis of GO and KEGG pathways in order to better understand the potential biological role of common DEGs. The results of KEGG assays showed that 140 upregulated genes were mainly enriched in p53 signaling pathway, Viral carcinogenesis, Transcriptional misregulation in cancer and Systemic lupus erythematosus (Figure 2A). The results of GO assays revealed that 140 upregulated genes were mainly enriched in spindle organization, spindle assembly, sister chromatid segregation and regulation of sister chromatid segregation (Figure 2B). The results of KEGG assays showed that 513 down-regulated genes were mainly enriched in cAMP signaling pathway, Vascular smooth muscle contraction, Tyrosine metabolism and Renin secretion (Figure 2C). The results of GO assays showed that 513 down-regulated genes were mainly enriched in response to steroid hormone, response to peptide hormone, response to ketone and response to glucocorticoid (Figure 2D).

### The Screen of Survival-Related Genes in BC

**FIGURE 3:**
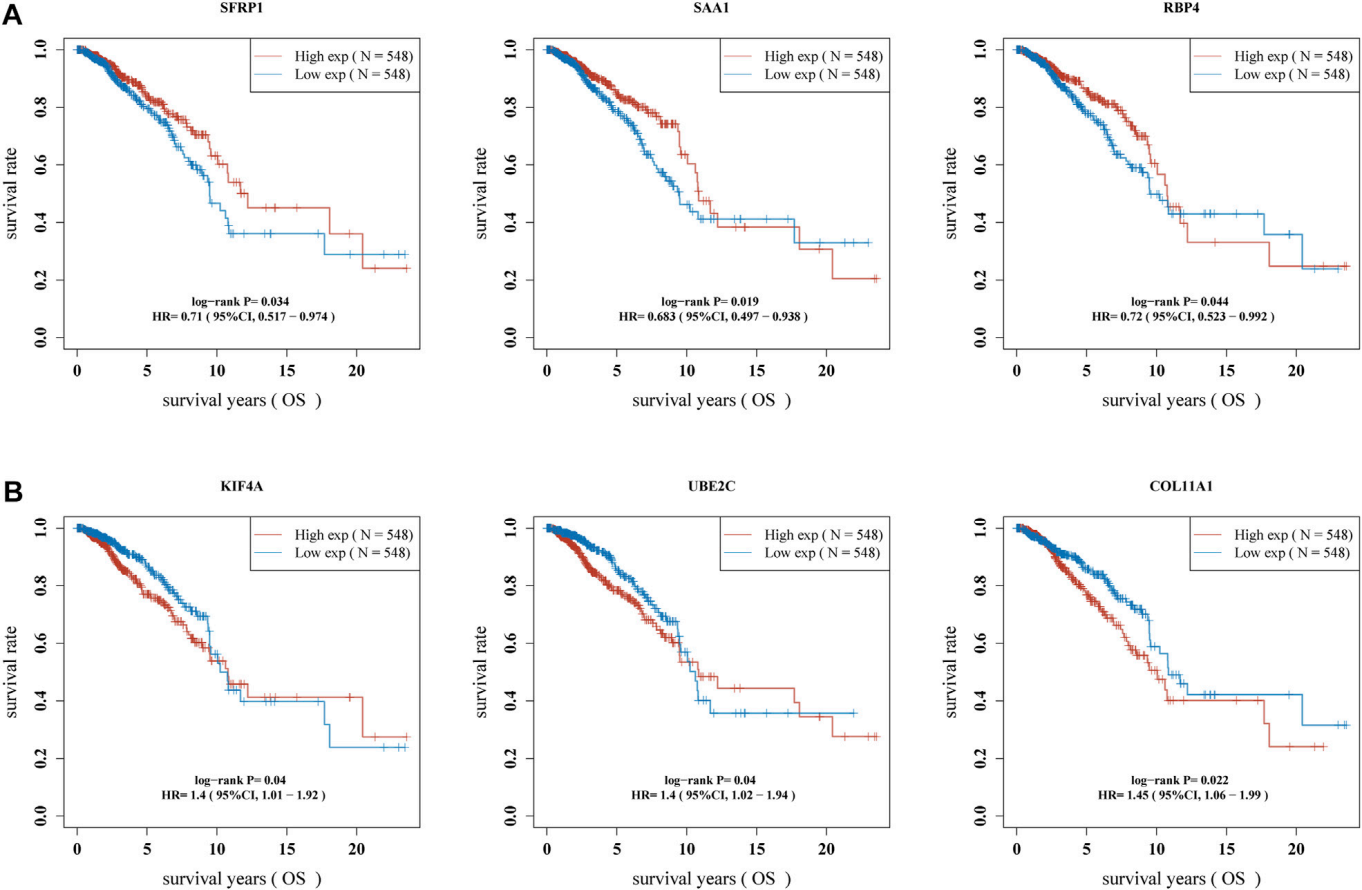
Identification of survival-related DGEs in BC patients. **(A)** high expression of SFRP1, SAA1 and RBP4 were associated with favorable long-term survival in BC patients. **(B)** High expression of KIF4A, UBE2C and COL11A1 was associated with poor prognosis in BC patients.

**FIGURE 4:**
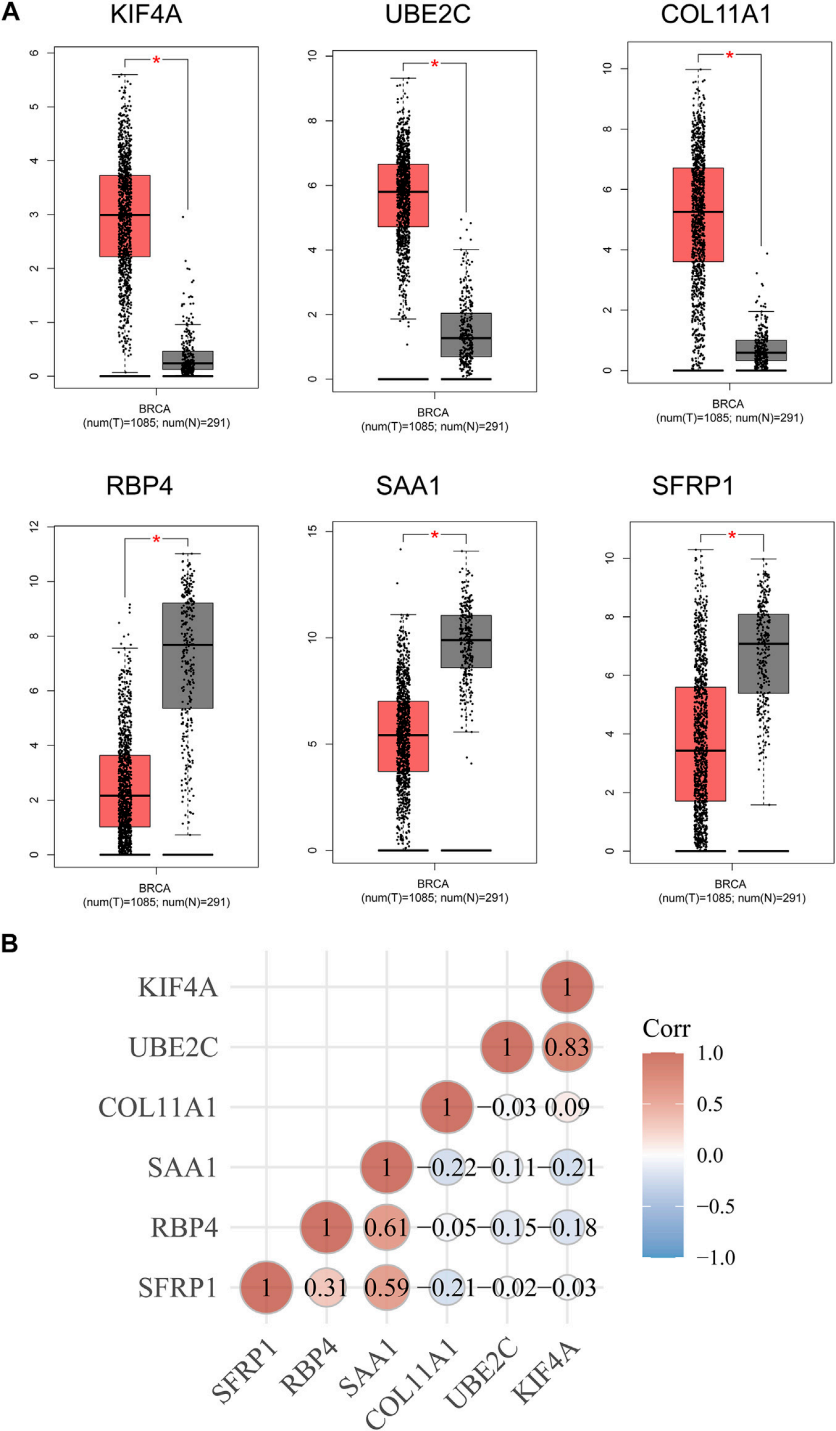
**(A)** The expression of the six survival-related genes in BC specimens and normal breast cancer specimens from TCGA and GTEx data. **(B)** The associations between the expressions of the six survival-related genes.

Then, we used Kaplan-Meier method to identify the survival-related genes using top 20 dysregulated genes in BC. As shown in Figure 3A, we found that high expressions of SFRP1, SAA1 and RBP4 were related to favorable long-term survival in BC patients, while high expression of KIF4A, UBE2C and COL11A1 was associated with poor prognosis in BC patients (Figure 3B). Moreover, we used GEPIA to further explore the expression of SFRP1, SAA1, RBP4, KIF4A, UBE2C and COL11A1 in both TCGA datasets and GTEx data. We confirmed that the expression of KIF4A, UBE2C and COL11A1 was distinctly increased in BC specimens compared with normal breast specimens, while the expression of SFRP1, SAA1 and RBP4 was distinctly decreased in breast cancer specimens (Figure 4A). The association among the six genes were shown in Figure 4B. There is a positive or negative association among them.

### Construction and Validation of a Prognostic Signature

**FIGURE 5:**
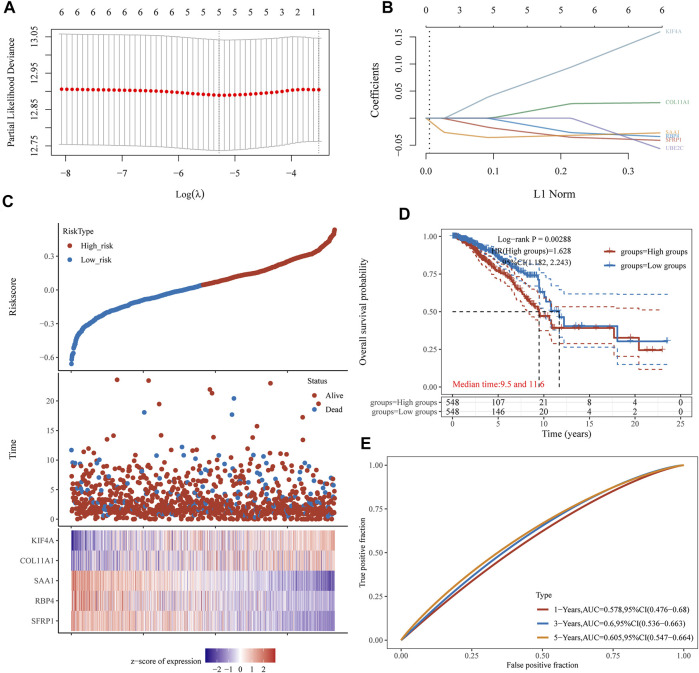
LASSO regression analysis of TCGA datasets identifies a five-gene risk profile for overall survival**. (A)** Adjustment of the proportional hazards model’s tuning parameters using cross-validation. **(B)** Scan of six BC genes with the LASSO coefficient spectrum. **(C)** Patient survival and BC status, as well as risk score distribution. **(D)** Kaplan-Meier was used to categorise patients based on their median risk of developing BC. **(E)** The risk signature’s predictive power was demonstrated using ROC curves.

**FIGURE 6:**
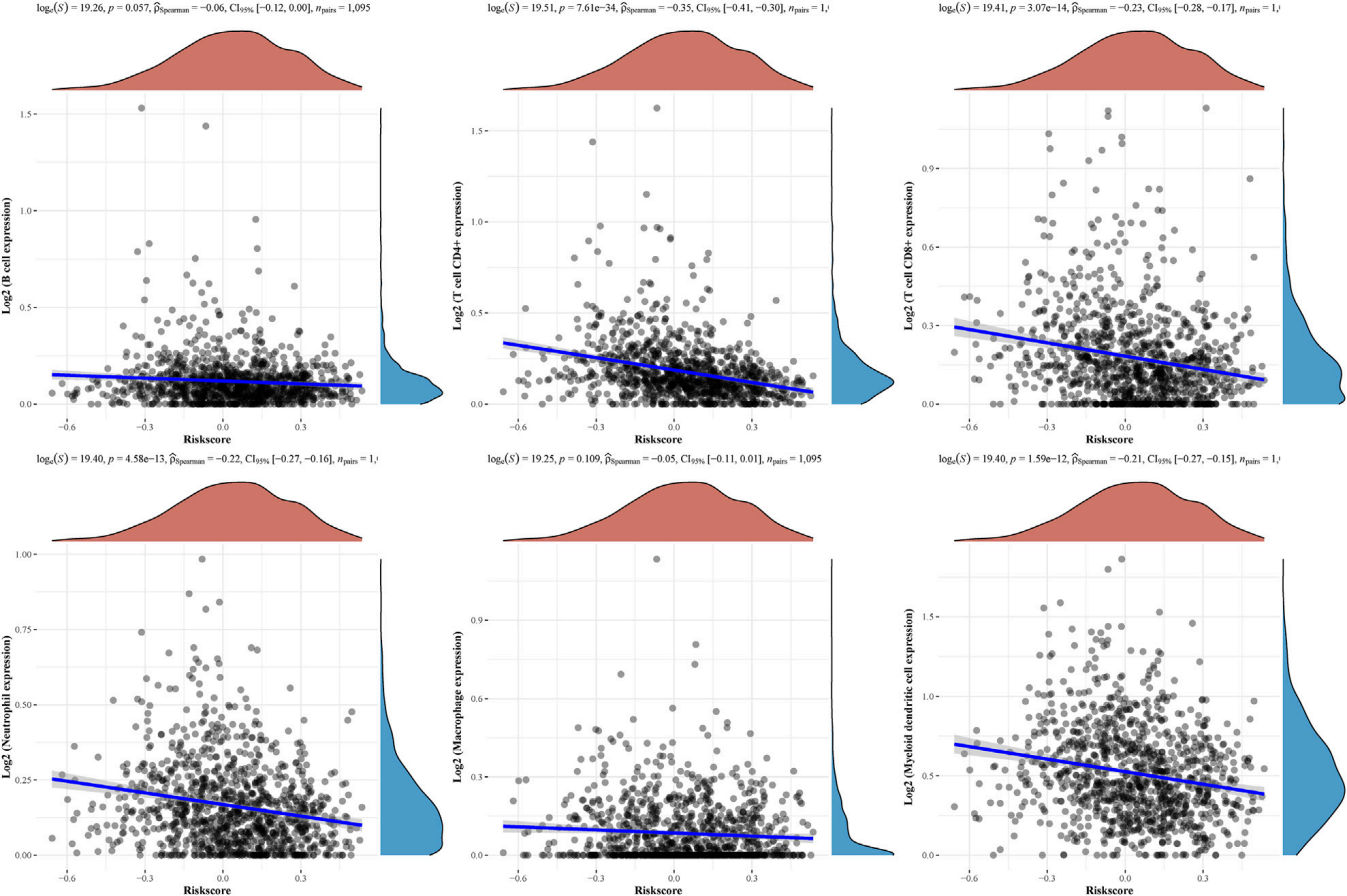
The relationships between the risk score model and immune cell infiltration were investigated based on TCGA samples.

Then, the above six genes were input into the LASSO regression model for feature selection. Under penalizing conditions (alpha = 1), five genes scores with nonzero coefficients were selected to formulate the risk score: Risk score = (–0.0305 × SFRP1 expression) + (–0.0194 × RBP4 expression) + (0.033 × SAA1 expression) + (0.019 × COL11A1 expression) + (0.0788 × KIF4A expression) (Figures 5A,B). The samples were separated into two categories based on the median risk score obtained from all LUAD samples: low-risk and high-risk groups. Figure 5C depicts a survival summary as well as a heatmap of gene expression levels in various tissues. According to the results of the survival analysis, patients in the high-risk group showed a distinctly shorter overall survival (Figure 5D). The area under the ROC curve for 1, 3, and 5 years OS were 0.578, 0.6 and 0.605 (Figure 5E). A study was conducted to determine the correlations between the risk score model and the presence of immune cells. As shown in Figure 6 B cell, T cell CD4^+^, T cell CD8^+^, Neutrophil, Macrophage and Myeloid dendritic cells were positively correlated with risk score. It has been confirmed that the levels of immune cells play an important role in the progression of various tumors (Sabado et al., 2017; Tanaka and Sakaguchi, 2019). Our findings further indicated the potential reason why our model was associated with the clinical outcome of BC patients.

### Knockdown of KIF4A Suppressed the Proliferation of BC Cells

**FIGURE 7:**
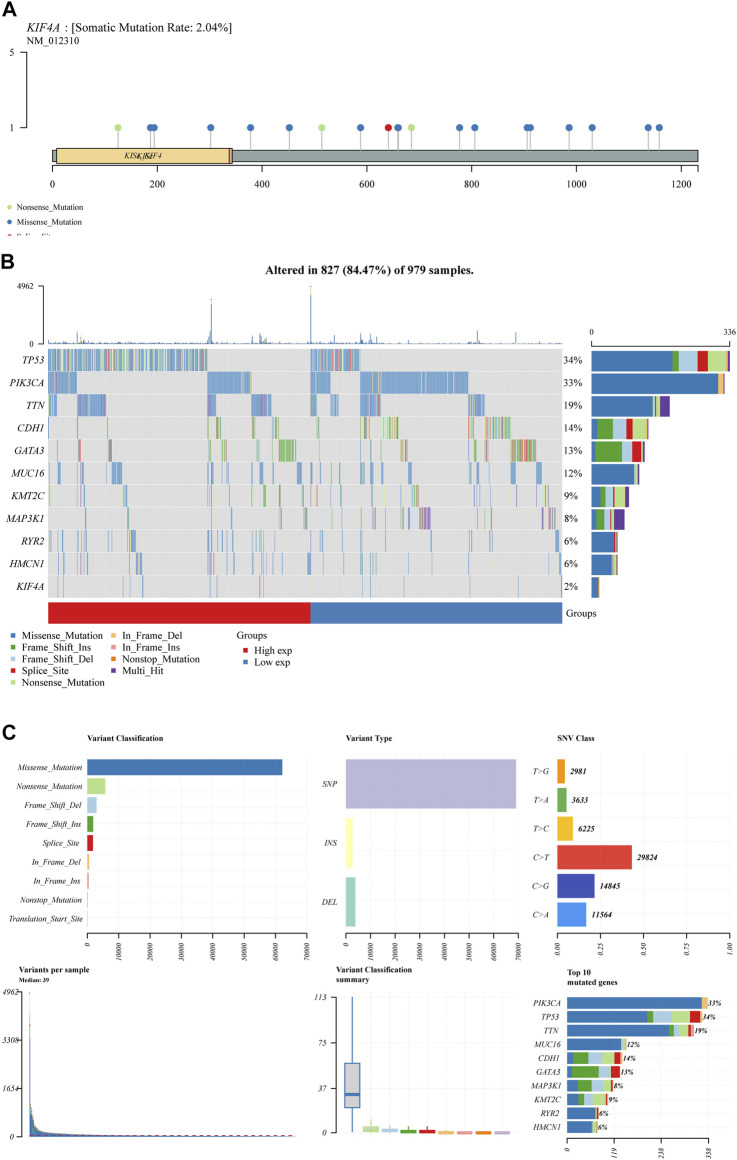
BC mutation cohorts in TCGA datasets. **(A,B)** Waterfall diagram depicting the TCGA BC cohort’s top 10 most frequently mutated genes, including KIF4A. **(C)** Overview of mutations in all BC samples.

**FIGURE 8:**
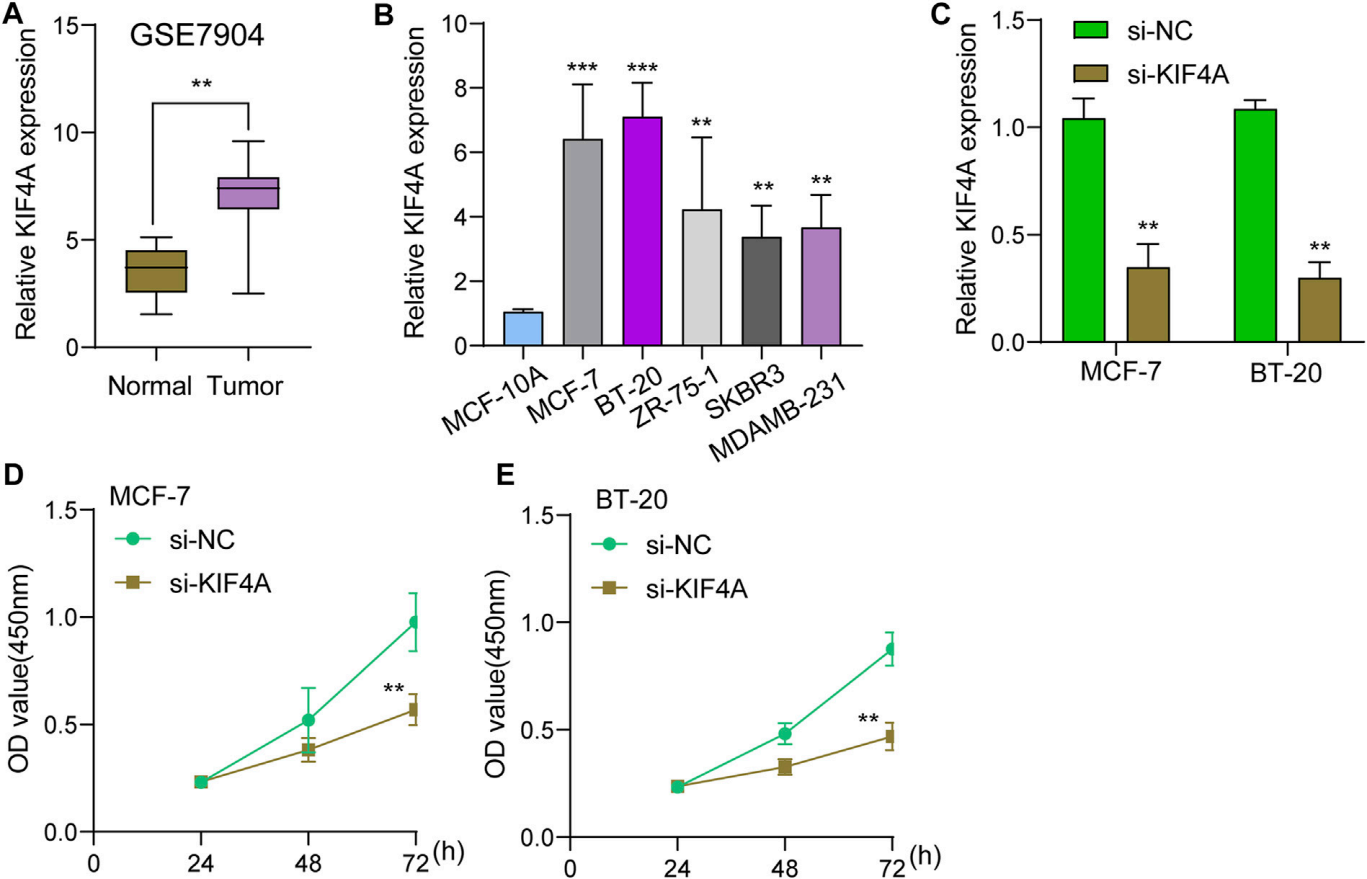
Knockdown of KIF4A suppressed the proliferation of BC cells. **(A)** The expression of KIF4A in BC sample and normal samples were determined using GSE7904 datasets. **(B)** Analysis of KIF4A gene expression in BC cell lines was carried out using qRT-PCR. **(C)** RT-PCR was used to examine KIF4A expressions in MCF-7 and BT-20 cells transfected with si-NC or si-KIF4A. **(D,E)** CCK8 assays for the assessment of the effect of KIF4A knockdown on the proliferation of MCF-7 and BT-20 cells. **p < 0.01, ***p < 0.001.

We assessed numerous basic aspects of BC somatic mutation data from the TCGA datasets using the waterfall and maftools analyses provided by the R package. As identified by a waterfall plot, the top 10 mutated genes were TP53, PIK3CA, TTN, CDH1, GATA3, MUC16, KMT2C, MAP3K1, RYR2, HMCN1, and the somatic mutation rate was also shown (Figures 7A,B). The summary plot exhibited that the main variant classification was missense mutation, It was discovered that SNP was the most prevalent type of variant, and that cytosine altered into thymine was the most common type of SNV class (Figure 7C). To further determine the expression of KIF4A in BC, we analyzed GSE7904, finding that KIF4A expression was distinctly upregulated in BC specimens compared with non-tumor specimens (Figure 8A). Then, we performed RT-PCR to examine the expression of KIF4A in several BC cells, finding that KIF4A expression was distinctly upregulated in Human BC cell lines (MCF-7, SKBR, BT-20, ZR-75–1, MDAMB-231) compared with MCF-10A cells (Figure 8B). By the use of si-KIF4A, we built KIF4A-knockdown cell lines (MCF-7 and BT-20, which was confirmed by RT-PCR(Figure 8C). Moreover, the results of CCK-8 assays revealed that knockdown of KIF4A distinctly suppressed the proliferation of MCF-7 and BT-20 cells (Figures 8D,E). Our finding suggested that KIF4A may influence the prognosis of BC patients *via* promoting the proliferation of BC.

## Discussion

Because of its complex molecular and cellular heterogeneity, BC is the most prevalent malignant tumour in women, accounting for one-quarter of all female cancer cases (Matsen and Neumayer, 2013; Fahad Ullah, 2019). Its incidence is increasing year after year, and it is the most common malignant tumour in women (Maughan et al., 2010). As a result, better understanding of BC biology may provide clinicians with new strategies to utilise in the treatment of the disease. Comprehensive genomic studies demonstrating the impacts of RNA have attracted a great deal of attention recently (Azim and Partridge, 2014; van 't Veer et al., 2002). A large number of potentially useful mRNAs must be identified in order to enhance the clinical outcomes of BC patients (Sun et al., 2019; Zhang and Yu, 2020). However, there is a limited number of particular markers that may be utilised to demonstrate therapeutic results, and prognostic criteria are significant in the management of BC patients. Thus, there is an urgent need for the identification of markers of BC in order to minimise mortality and improve the prognosis of cancer patients.

Using the TCGA database, we examined the gene expression variations between BC and normal breast tissues in this work in order to discover possible gene biomarkers. After screening DEGs, Lasso analysis was applied to build a risk model for predicting the prognosis of BC. We identified five genes: SFRP1, RBP4, SAA1, COL11A1 and KIF4A. high expressions of SFRP1, SAA1 and RBP4 were related to favorable long-term survival in BC patients, while high expression of KIF4A and COL11A1 was associated with poor prognosis in BC patients. In addition, patients in the high-risk group exhibited distinctly lower overall survivals, demonstrating that the four-gene signature had a good ability to predict mortality.

Various cell types are critical to tumour immunology, and the tumour microenvironment (TME) is a fundamental component of cancer (Ren et al., 2021). The response to immunotherapy may be influenced by the TME infrastructure and the interactions between cancer cells and TME throughout the onset and course of the disease (Crespo et al., 2013; Sun et al., 2018). As part of the tumour stroma, tumor-infiltrating immune cells play a key role in tumour progression and response to cancer therapy (Certo et al., 2021; Singleton et al., 2021). Researchers looked into the connections between the risk score model and immune cell infiltration. We found that B cell, T cell CD4^+^, T cell CD8^+^, Neutrophil, Macrophage and Myeloid dendritic cells were positive correlated with risk score, suggesting the importance of our signature in the immune system.

Secreted frizzled-related protein 1 (SFRP1) belongs to the secreted glycoprotein SFRP family (Baharudin et al., 2020; Cisneros et al., 2020). Since SFRP1 has been found to be down-regulated in a number of human malignancies, it has been designated as a tumour suppressor gene. This is mostly due to epigenetic inactivation by DNA methylation or transcriptional silence by miRNAs (Zhang et al., 2019; Sunkara et al., 2020). SFRP1 protein expression has been shown to be closely linked to BC, according to one study (Veeck et al., 2006; Schäfer et al., 2019). SFRP1’s usefulness as a biomarker for chemotherapy response in BC is supported by associations with age and tumour grade (Gregory et al., 2019).

Retinol binding protein 4 (RBP4) is a 21-kDa protein belonging to the lipocalin family and is a retinol transporter in the blood (Steinhoff et al., 2021). Growth, eyesight, and metabolic disorders are all impacted by RBP4, an adipokine mostly produced in the liver and fat (Wang et al., 2018; Zhao et al., 2021). In recent years, several studies have reported that RBP4 was dysregulated in several types of tumors (Fei et al., 2017; Karunanithi et al., 2017). However, the function of RBP4 was rarely reported in BC.

SAA1 protein belongs to a member of the serum amyloid A family of apolipoproteins (Jiang et al., 2021). An important acute-phase protein known as SAA1 is increased in response to inflammation and tissue injury (Zhou et al., 2019; Gan et al., 2020). Besides, suppression of SAA1 expression can also occur after surgery or in late cancers. The prognostic value of SAA1 in BC has been frequently reported (Cao et al., 2021; Olivier et al., 2021).

Kinesin family member 4A (KIF4A), a KIF protein, is an essential chromosome-associated molecular motor encoding a 140-kDa protein (Cuijpers et al., 2020). KIF4A has been implicated in the regulation of chromosomal condensation and segregation, middle-spindle formation, and mitotic cytokinesis, according to previous research. Further researches have shown that KIF4A operates as an oncogene and plays critical roles in a number of malignancies, including breast cancer, prostate cancer and colorectal cancer (Matsumoto et al., 2018; Xue et al., 2018; Cao et al., 2020). In this study, we analyzed BC somatic mutation data from the TCGA database, and found that KIF4A showed a high level of somatic mutation. Then, we chose it for further study. Based on the results of GSE7904, we further confirmed that KIF4A expression was distinctly upregulated in BC specimens. The results of RT-PCR also confirmed that KIF4A expression was highly expressed in BC cells, which was consistent with the results form TCGA datasets. We further explored its function, finding that knockdown of KIF4A distinctly suppressed the proliferation of BC cells, suggesting that it acted as a tumor promotor in BC progression.

Several limitations existed in our study. First, Because the sample lacked certain clinical follow-up information, we were unable to identify predictive biomarkers based on criteria such as the existence of other health disorders. Secondly, bioinformatic approaches using RNA-seq data revealed the immunological landscape. Noise may have affected this evaluation. Thus, a larger number of participants in the experiments, along with additional genetic testing, will be needed in the future.

## Conclusion

Here, A collection of biologically significant genes and a five-gene signature that has been independently validated have been developed using integrated studies. Hopefully, our 5-gene signature may be a clinically beneficial tool for individualized treatment of BC.

## Data Availability

The datasets presented in this study can be found in online repositories. The names of the repository/repositories and accession number(s) can be found below: https://portal.gdc.cancer.gov/, TCGA https://www.ncbi.nlm.nih.gov/, GSE7904.
